# Management of the Acute Diarrheas of Infancy

**Published:** 1900-06

**Authors:** Gilman P. Robinson

**Affiliations:** Lecturer on Diseases of Children, Atlanta College of Physicians and Surgeons


### MANAGEMENT OF THE ACUTE DIARRHEAS OF INFANCY.

By GILMAN P. ROBINSON, M.D.,

Lecturer on Diseases of Children, Atlanta College of Physicians and Surgeons.

The diarrheas of infancy may be divided, on the grounds of etiology, into two classes. First, those of nervous origin, and second, those caused by bacteria. D'Orlandi has shown that while in the feces of the former there are a great many bacilli, yet there are a very small number of cocci, with only an occasional diplococcus and never streptococci; whereas in the latter form the diplococci were very numerous, and in a severe case of gastro-enteritis two chains of streptococci were found.

### SIMPLE DIARRHEA.

The diarrheas of nervous origin are the result or manifestation of increased peristalsis. This increased peristalsis may be caused by different conditions acting through the central nervous system or by the mechanical action of undigested food. The most important examples of the first are those caused by sudden changes in temperature, long exposure to heat or cold, fright or fatigue; of the second, food that has failed of digestion either on account of its unsuitableness for digestion or because the digestive power is functionally weak. In either case such food acts as a foreign body. The mucous membrane of the intestine shows no pathological changes. The number and consistency of the dejections are increased, and, except for the presence of undigested food, they are normal in character. Possibly as good a name as any to call these diarrheas are "simple diarrheas." Their chief danger lies in the fact that they render the intestinal tract liable to the invasion of micro-organisms. For this reason they never should be neglected, especially in warm weather.

### TREATMENT OF SIMPLE DIARRHEA.

The treatment consists essentially in removing the cause. In case of fatigue or fright, it is an easy matter. When caused by temperature, a change of climate may be necessary. When the trouble is caused by undigested food, it, of course, should be gotten rid of. This is best accomplished either by castor oil or calomel. In most cases castor oil is preferable, giving it to infants under one year of age in two teaspoonful doses. If there is vomiting calomel is better, and should be given in 1-10 grain doses combined with bicarbonate of soda every half hour until a grain is taken. The infant should be kept quiet. Rarely, it may be well to wash out the stomach and colon.

As a rule the diarrhea ceases after the cause has been removed; if, however, it does not cease, then attention should be given the increased peristalsis. Opium, in the shape of paregoric or some of the astringents, can be guardedly used. This condition, however, with very rare exceptions, is the only condition in which the use of opium is allowable. It is generally well to withhold food for several hours at the beginning, and then to begin with a dilute alkaline sterilized milk. Brandy, at times, is very efficacious, as the increased peristalsis is often due to a general weakness.

INFECTIVE DIARRHEA.

Micro-organisms are the cause of a large proportion of the acute diarrheas of infancy. These organisms may be the ordinary cocci found in the intestines, or they may be such as are not ordinarily found there. They, as a rule, enter the body with the food. Large numbers of examinations, however, have failed to show any relation between any special form of bacteria and variety of diarrhea. Hence our present knowledge of the bacteriology of the discharges give us no help either in diagnosis or in treatment. The intestines may show all grades of inflammation from simple catarrhal or simple follicular hyperplasia to follicular ulceration and the formation of false membrane. The severity of the symptoms are often no index as to the extent of the pathological changes.

The constitutional symptoms are due partly to lack of nourishment and the withdrawal of fluids; but chiefly to the absorption of the toxins. The most common of the symptoms due to toxic absorption are reflex vomiting, hyperpyrexia, prostration with cardiac depression and nervous excitement shown in restlessness, incessant motion and sleeplessness.

The acute diarrheas due to micro-organisms may be roughly divided, using the nomenclature of the American Pediatric Society, into the fermentative diarrhea, ileocolitis and cholera infantum; all calling to a certain extent for different lines of treatment, yet certain general methods being applicable to all. The most essential is prophylaxis.

### PROPHYLAXIS.

Prophylaxis, while important at all times, is particularly so during the summer and early fall. The very best hygienic surroundings should be thrown around the infant; it should be given plenty of fresh air and bathed often. Care should be used not to over-feed, as less food is required during the hot weather, and again depression caused by the heat retards digestion. A great deal of pure water should be given to make up this deficiency. As these micro-organisms are introduced with the food, great care should be used to always give a fresh and sterile food, and especial care should be given to the milk supply. It should be remembered that pasteurization or sterilization of milk does not destroy the toxins in it; it merely prevents the formation of more. The toxins present in milk before it is sterilized may be sufficient to cause severe or fatal symptoms.

Hygienic measures are of great importance in treatment as well as in prophylaxis. The infant should be kept quiet, given as much fresh air as possible, bathed often and lightly clothed, protecting, however, the abdomen with either a spice bag or warm flannel. The napkins should be disinfected at once to prevent reinfection, since, as has been stated, many of the most serious symptoms are due to the absorption of the toxins; hence it is evident that any treatment that tends to prevent the natural elimination of these poisonous products in the dejections is absolutely *bad*. The frequent discharges are nature's method of elimination and attempt to cure. Therefore, opium in any form is positively contraindicated. It can but simply do harm. Astringents are only less harmful because they are less active.

### FERMENTATIVE DIARRHEA.

Almost all the summer diarrheas belong to the fermental class, but in making a diagnosis on the ground of symptomatology, it should be borne in mind that in many cases there is no relation between the severity of the symptoms and that of the pathological lesions, and that conclusions based entirely on symptoms may be erroneous.

The number of discharges in fermental diarrhea is only moderate, as a rule not more than ten or twelve in twenty-four hours. The color varies from yellow and green, through all the shades of green. Often they are watery or frothy and may contain more or less curds and mucus, but rarely blood. The amount of the discharge is usually comparatively large. The odor is always offensive,—in some cases sour, and in others very foul. On this basis there have been two classes recognized: the acid fermentation (the sour), and the albuminoid decomposition (the foul odor). In the first class the trouble is supposed to be due to the bacteria which thrive on the sugar or fats, and in the second to those which thrive on the proteids. Tenesmus and pain are not as common as in ileocolitis. The various constitutional symptoms are present in all cases in various degrees of severity. Numerous conditions are met with, from the mild cases lasting a few days or weeks, to the severe toxic conditions fatal in a few days or even hours.

### TREATMENT OF FERMENTAL DIARRHEA.

The first indication is to remove the cause viz.: the bacteria causing the trouble and their toxic products. Castor oil is, I think, usually the most satisfactory when there is no vomiting, though calomel is always applicable in all cases, especially when there is vomiting. If the vomiting is severe and continues, it is well to wash out the stomach. This is a simple matter in infants, using a soft rubber catheter, cutting off the tips, and rounding the edges by heat. A normal salt solution or a solution of borax or bicarbonate of soda are better than simple sterile water. If there is intense thirst it is well to leave a few ounces in the stomach.

It is generally well to wash out the colon. This is done by using a rather large soft rubber catheter and attaching it to a fountain syringe not more than three feet above the patient. This catheter should be introduced high up into the bowel and several quarts of sterile water, or better, sterile water with one drachm of borax to the pint, allowed to run in and out. Tepid water is preferable. The value of washing both ends of the alimentary tract can hardly be overestimated.

Unfortunately, however, lavage of the alimentary tract does not rid it entirely of bacteria. Enough remain, if the conditions for their multiplication are favorable, to keep up or cause a reinfection. A condition most favorable for their reproduction is some product in the intestine which is a suitable culture medium. This medium enters the intestine through the mouth ; therefore, our next indication for treatment is not to supply any such medium. For this reason it is best to stop all food for twenty-four hours. The praises for barley water, so often heard, are undoubtedly due to the fact that it contains little besides water, so that its use practically amounts to starvation.

Again, unfortunately for us, starvation does not diminish the number of bacteria to a point where they can do no harm, and besides the child demands nourishment. It must be given the least harmful form of food, or the form least liable to act as a culture medium. The odor of the stools as being characteristic of acid fermentation or albuminoid decomposition would seem to be some indication. Unfortunately, however, with the exception of the sour stools, this distinction is seldom possible. Here an albuminoid diet with an almost complete elimination of sugar certainly gives satisfactory clinical results. Ordinarily, however, the process seems to be a more complex one, and the selection of the food must be done on general principles.

Foods that are digested high up in the alimentary tract seem the most suitable. Personally I have found egg albumen and Fairchild's panopepton very useful. Another food I like very much, though I have never seen it advocated, is okra given in the form of a carefully strained soup. There seems to be a general opinion that as in the milk the bacteria are introduced into the system, it makes a good culture medium, and hence milk should not be used as a food. The ordinary custom seems to be to give some form of starchy food. I cannot see why if a starchy food is unsuitable for a well baby, it should be any less bad for a sick one. After the preliminary starvation I have always had good results in feeding a weak modified mixture of absolutely fresh milk. It is, without doubt, due to the dilution and freshness of the milk that the favorable clinical results are obtained. The mixture should be prepared from an absolutely fresh milk, and should be low in all its percentages, highly alkaline and pasteurized or sterilized. Experience will teach what percentages to give in each individual case. It is better to give small feedings often than large ones at long intervals. As the patient improves, the percentages may be gradually increased, together or singly, and the intervals lengthened until the proportions and intervals are such as are given to a normal infant of that age.

One of the ways of removing the cause is to destroy the cause, but unfortunately, in these cases the destruction of the cause involves the destruction of the patient. Germicidal drugs sufficiently strong to be efficacious cannot be used with safety. Drugs, however, which limit the activity of the organisms can be used to advantage. After a considerable and varied trial of many of the antiseptic drugs, and many of the newer so-called intestinal antiseptics, I have given up all except bismuth. I would say here, that this also is the general consensus of opinion of all those who have much to do with children. Bismuth is best given as the subgallate or subnitrate. Either, however, to be effectual must be given in large doses, not less than from one to two drachms in twenty-four hours. It is best given as a powder or in suspension in water or milk.

The sulphocarbolate of zinc in doses of $\frac{1}{6}$ to $\frac{1}{4}$ of a grain seems to help the formation of gas and to improve the odor.

Stimulation is necessary under the same conditions as are met with in other diseases.

Certain conditions due to toxic absorption may require special treatment. Excessive vomiting is best treated by washing the stomach, fever by baths, extreme restlessness usually yields to warm sponge baths or the bromides. In prostration and collapse stimulants are to be given along the usual lines. Brandy and strychnine are the most useful, and as quick action is often necessary, they are better given under the skin.

### ILEOCOLITIS.

In ileocolitis are found the more serious pathological lesions. They are more marked in the colon and lower part of the small intestine. The discharges are usually small in amount, and contain much mucus, blood and sometimes pus and membrane. The fecal matter is generally pretty well digested. The odor varies, being putrid when there is sloughing and deep ulceration, but almost nil when there is much mucus.

Pain and tenesmus are marked symptoms. The abdomen, while soft at first, is often tender and distended. Vomiting is usually not a prominent symptom.

### TREATMENT OF ILEOCOLITIS.

The treatment is along the same general lines as the fermentative diarrheas. Purgation, starvation and diet are important in both conditions. The proteids are better borne in ileocolitis than in the other forms, and should constitute a large proportion of the diet. The fats, however, are not so well borne, and should be given in very low percentages. Bismuth is even more useful in this condition, as it not only diminishes the fermentation but also mechanically coats over the inflamed surfaces.

As the location of the lesions are usually low down it is possible to reach the seat of the disease from below by irrigation. This provides a means for not only washing out the micro-organisms, but also of making local applications to them. Several quarts should always be used, and should be allowed to run until it returns clear. Normal salt solution or a two to four per cent. boracic acid solution is the best. Stronger solutions of antiseptics may be dangerous and are no better.

Irrigations should be used two or three times daily. A solution of starch water with one or two drachms of subnitrate of bismuth suspended in it should be injected after each irrigation. In this way the drug may be directly deposited upon the inflamed portion. Suppositories of $\frac{1}{10}$ or $\frac{1}{6}$ of a grain of cocain are often very efficacious for the tenesmus. Other special treatment is the same as in the fermentative diarrheas.

### CHOLERA INFANTUM.

It is a common practice of many physicians of calling all severe cases of either fermentative diarrheas, or ileocolitis, cholera infantum. The term should be applied only to those cases characterized by the most severe cholera form symptoms. It is a very rare condition. The pathological conditions are slight, never progressing further than hyperemia and desquamative catarrh.

Vomiting and diarrhea are constant, and both very soon become serous not mucous. As a consequence the tissues are rapily deprived of their fluids. Thirst is very marked. Wasting and prostration is extreme. The internal temperature is high, though the surface temperature may be low. There is internal congestion. There are very marked nervous symptoms. It is a self-limited disease, and its course is not more than two or three days, usually less. The termination is almost always death.

### TREATMENT OF CHOLERA INFANTUM.

It is self-evident that in so rapid and fatal a disease the treatment must be both quick in its action and vigorous. There is probably vasomotor paralysis of the gastro-intestinal vessels. Hence food and drugs introduced into the intestinal canal cannot be absorbed, and thus can do no good and may do harm.

There are five main indications for treatment: (1) To empty the stomach and bowels of their toxic contents, (2) to supply fluid to the depleted tissues, (3) to restore the surface circulation, (4) to reduce the internal temperature, (5) to sustain the strength until the disease has run its course.

Purgatives act too slowly; hence our chief reliance must be in washing the stomach and irrigation of the lower bowel. This must be done early and vigorously. It is useless to try to supply fluids by the mouth, as they will be immediately vomited. Cold sterile water can be tried, however. The injection of normal salt solution into the cellular tissues is the only method of introducing fluid that can be depended upon. A pint should be given during the twenty-four hours. This not only supplies fluid to the tissues but helps to eliminate the toxic products and to restore surface circulation.

Irrigation of the lower bowel with cold water not only tends to lessen the internal temperature, but restores the surface circulation. The best methods for doing this, however, are rubbing, mustard baths, and the warm pack. These procedures, however, are not those best fitted to reduce the temperature, hence it is well to use cold in the form of sponging, packs, and baths. Often it is necessary to determine in individual cases whether the internal congestion or the temperature is doing the most harm, and to treat the most serious condition. The relief of one often helps the other.

As food cannot be given by the mouth, the patient must be kept alive by stimulation. As drugs given by the mouth are not absorbed, this stimulation must be given subcutaneously. The usual stimulants are given, though atropine seems to be the most useful. It should be given in doses of one five-hundredth to one eight-hundredth of a grain. Morphine can be given where the diarrhea, vomiting or restlessness are extreme. One one-hundreth of a grain is usually sufficient. Give it under the skin, and use care not to give it too long nor in too large doses.

As imprcvement begins, stimulants and water can be given by the mouth, and soon pasteurized milk of very low percentages, can be added.

